# Evaluation of Shear Strength of RC Beams with Multiple Interfaces Formed before Initial Setting Using 3D Printing Technology

**DOI:** 10.3390/ma10121349

**Published:** 2017-11-24

**Authors:** Kyeongjin Kim, Sangmin Park, WooSeok Kim, Yoseok Jeong, Jaeha Lee

**Affiliations:** 1 Department of Civil and Environmental Engineering, Korea Maritime and Ocean University, 727 Taejong-ro, Yeongdo-gu, Busan 49112, Korea; kkj4159@naver.com (K.K.); psm9153@naver.com (S.P.); 2 Department of Civil Engineering, Chungnam National University, 99 Daehak-ro, Yuseong-gu, Daejeon 34134, Korea; wooseok@cnu.ac.kr (W.K.); yosoksi@gmail.com (Y.J.); 3 Department of Civil Engineering, Korea Maritime and Ocean University, 727 Taejong-ro, Yeongdo-gu, Busan 49112, Korea

**Keywords:** layered concrete, fracture energy, shear strength, initial setting

## Abstract

With the recent development of 3D printing technology, concrete materials are sometimes used in 3D printing. Concrete structures based on 3D printing have been characterized to have the form of multiple layer build-up. Unlike general concrete structures, therefore, the 3D-printed concrete can be regarded as an orthotropic material. The material property of the 3D-printed concrete’s interface between layers is expected to be far different from that of general concrete bodies since there are no aggregate interlocks and weak chemical bonding. Such a difference finally affects the structural performance of concrete structures even though the interfaces are formed before initial setting of the concrete. The current study mainly reviewed the changes in fracture energy (toughness) with respect to various environmental conditions of such interface. Changes in fracture energies of interfaces between concrete layers were measured using low-speed Crack Mouth Opening Displacement (CMOD) closed loop concrete fracture test. The experimental results indicated reduction in fracture energy as well as tensile strengths. To improve the tensile strength of interfaces, the use of bridging materials is suggested. Since it was assumed that reduction in fracture energy could be a cause of shear strength, to evaluate the reduced structural performance of concrete structure constructed with multiple interfaces by 3D printing technology, the shear strength of RC beam by 3D printing technology was predicted and compared with that of plain RC beam. Based on the fracture energy measured in this study, Modified Compression Field Theory (MCFT) theory-applied Vector 2 program was employed to predict the degree of reduction in shear strength without considering stirrups. Reduction factors were presented based on the obtained results to predict the reduction in shear strength due to interfaces before initial setting of the concrete.

## 1. Introduction

In recent years, 3D (three dimensional) printing technology has gained large attention and has become important in many engineering applications. In this technology, powdered forms of materials such as resin and metals are processed to produce 3D shapes very precisely based on design data. In 2013, global management consulting firm McKinsey selected 3D printing technology as one of the 12 technologies that are expected to bring innovation [1]. Typically, in construction industry, the size of construction forms is larger than that in other industries. Compared to other industries, the construction sector has a very low ratio of plant-produced elements (forms of combining diverse parts and materials instead of automation). Since a huge capital is necessary for construction automation and others, it is not easy to apply this 3D technology in the construction sector. On the other hand, it is also expected that 3D printing technology would help solve many problems encountered in the construction industry, such as low labor efficiency, high accident rate (loss of lives) and too much manpower necessary for construction site control and surveillance [2]. It was also reported that 3D printing technology could reduce the amount of CO_2_ and construction wastes compared to the conventional way of construction by 75% and 86%, respectively [3]. However, many studies are still necessary for the application of 3D printing in large-scale civil structures. Concrete-based 3D printing technology has been applied in mid- to smaller-sized structures, such as houses or aesthetic structures. 

**Figure 1:**
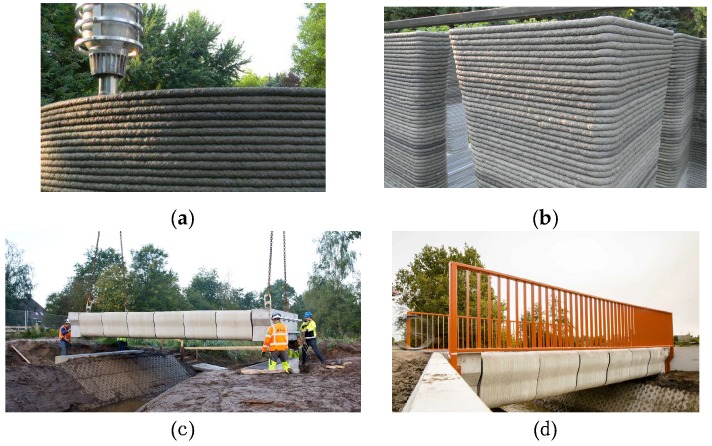
3D printing technique using concrete material: (**a**) Rudenko (2014); (**b**) Rudenko (2014); (**c**) Salet, TU/e (2017); (**d**) Salet, TU/e (2017).

As shown in Figure 1a,b, to build a 3D concrete structure, 3D printing technology was applied so that each layer was built on top of the other by spreading them out through nozzle [4]. A detailed information on building up each layer through 3D printing technology could be found in literature Kwon (2002) [5]. Using this technique many interesting structures have been formed which exist till now. For example, 3D printed bridges for bike were designed and now open to public as shown Figure 1c,d [6]. Other structures such as an office in Dubai in UAE and Lewis Grand Hotel in Philippines were also constructed by building up layers through 3D printing technology. Other methods, such as powder-based 3D printed cement, require a certain form of base-structure made by steel or fabric for applying cement powder [7]. Recently introduced carbon textile-reinforced mortar could be a base-structure for powder or mortar based 3D printing technology [8]. In the present study, our aim was to build layers of concrete without using any mold.

In a multilayered structure, material properties (e.g., splitting tensile strength and fracture energy) of interlayer interface are expected to be weaker than those of normal concrete bodies. Due to these weak material properties of layer interface, a proper stacking plan should be implemented in the construction process to achieve interface integrity, strength and durability. The material properties of interface between the concrete layers depend upon the water-cement ratio, concrete viscosity, elapsed time to meet new layer, and superimposed dead load from upper part. Additionally, crack formation phase due to temperature, humidity, creep, and drying shrinkage, is also expected to affect the material properties of the interface.

**Figure 2:**
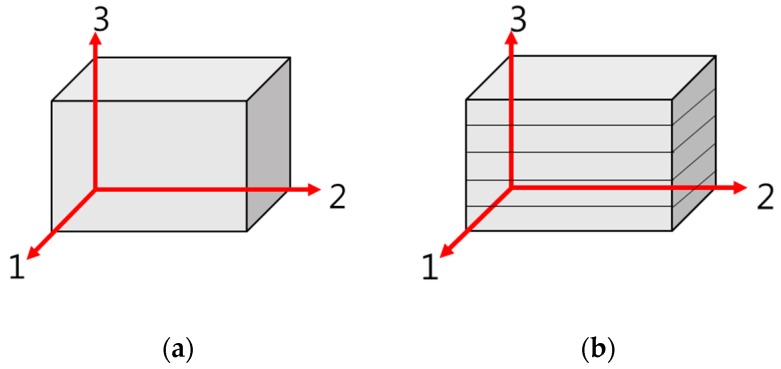
Normal concrete (homogeneous material) and 3D printed concrete (orthotropic material): (**a**) homogeneous material; and (**b**) orthotropic material.

As shown in Figure 2, concrete walls built using 3D printing technology are classified under orthotropic structures where multiple interfaces are layered in parallel, unlike normal concrete structures which are usually assumed homogeneous.

In the present research, a low-speed Crack Mouth Opening Displacement (CMOD) closed loop fracture tests were conducted based on concrete fracture mechanics to study reduction in concrete fracture energy due to addition of interfaces between concrete layers. As environmental conditions, time to meet new layer was considered because the longer the time to meet new layer, the larger is the reduction in interface strength and fracture energy due to curing of the concrete. The other conditions were kept unaltered throughout the experiments. 

The current study also suggested the use of bridging material to recover and compensate the reduced concrete integrity. To evaluate the strength reduction in concrete structures with several interfaces, shear strength of RC beam with several interfaces was estimated. Based on the fracture energy measured in the experiment, Vector 2 program was utilized to estimate the degree of reduction in shear strength due to formation of interfaces between concrete layers. It was assumed that formation of interfaces occurred before initial setting time. The obtained results could be useful information for estimating the strength of concrete structure built via 3D printing technology.

## 2. Experimental Setup for Evaluating Fracture Energy

This study established an experimental plan to create a set of 3D printing constructional conditions for specimens in an indirect way. The production method is described in more detail in later Section 3. 

Selected cement is Type I Portland cement and slump of the concrete used was measured at 130 mm (target value: 100 mm). The average 28-day compressive strength was 31.85 MPa; air volume, 4%; and maximum size of aggregate, 25 mm. The concrete from same batch was used to implement experiment on time for stacking. Change in fracture energy was observed according to various environmental conditions. To estimate the bilinear tension softening diagram (stress and crack width), the final form of load-MOD graph from fracture energy test and concrete tensile strength from splitting tensile test are also necessary. 

**Figure 3:**
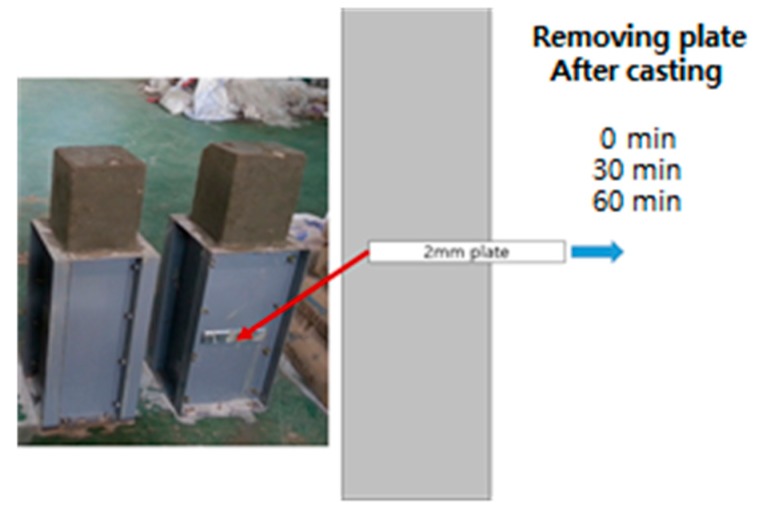
Formations of interface at different times.

To estimate fracture energy, compression strength test, three-point bending test and splitting tensile test were conducted. For the compression strength and splitting tensile tests, regular 10 cm-diameter and 20 cm height cylindrical specimens were used. The three-point bending test used specimens having 150 mm square cross section and 450 mm span length (total length: 550 mm). To induce cracks to the center, a notch which has 1/3 height of the beam was used, as shown in Figure 3. 

In this study, to implement low speed CMOD control experiments, high-precision clip on gauge (EPSILON 3541-005M-100M-ST) was employed. The measuring device is a high-precision device with 1.5 μm resolution and maximum nonlinearity of 0.064% during a total of 10 mm. The load cell of the equipment used was initially 1000 kN and it was replaced with Instron’s maximum 50 kN small load cell to increase load measuring precision by allowing load measurement in 20% (5–10 kN) of maximum load measuring capacity. The servo valve was also replaced with a small 1 GPM (94 LPM) capacity, a high-performance pressuring device made by JKS, for precise control in this tests. To receive and process CMOD signals from outside, an additional internal algorithm was designed to connect to the equipment controller since this CMOD control is not a built-in function in the controller (DAQ). As such, CMOD control fracture toughness test of concrete requires many considerations for experimental setup preparation and the actual experiment procedures. There is a study finding that simple stroke control included in most of the hydraulic machine supports final fracture energy measurement reliability instead of using CMOD control [9]. However, for more reliability in study findings rather than efficiency, all of the present study experiments were implemented using the CMOD control presented by ACI 446 committee [10]. 

## 3. Elapsed Time to Meet New Layer and Bridging Materials

As in Figure 3, to consider the elapsed time to meet new layer (accumulation time), plates were placed inside during concrete pouring to artificially prevent two layers from contacting each other. The pre-installed plates were removed after 0, 15, 30 and 60 min, as shown in Figure 3. Even though specimens were not formed by actual 3D printing technology, it was assumed that these procedures indirectly mimic the interface since the aggregates are aligned within a layer and did not cross to the other layer. This means that surface of layer is mainly smooth. When concrete is extruded by actual 3D printing machine pressure through a nozzle, surface will be smooth and coarse aggregates are normally trapped within layers.

**Figure 4:**
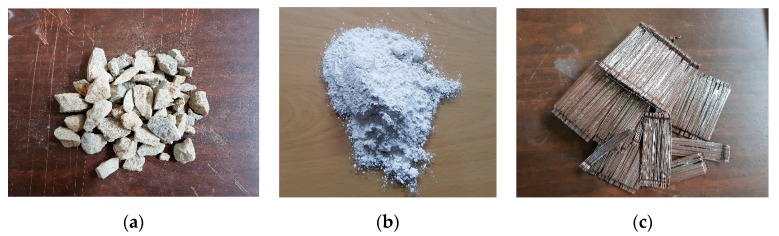
Selected bridging materials: (**a**) crushed aggregate; (**b**) retarder; and (**c**) steel fiber.

Table 1 shows specimen types according to different experimental conditions. Additionally, different bridging materials for enhancing interface integrity have also been considered. To improve the structural integrity of 3D printed concrete structures, three bridging materials were considered, as shown in Figure 4. The bridging materials used in the present study are steel fiber, aggregates, and retarder. Selected type of steel fiber has 60 mm length and diameter of the steel fiber is 0.9 mm. Yield strength provided by manufacturer is 700 MPa. For aggregates, crushed type with maximum size 25 mm was selected to take advantage of irregular shapes as a bridge material. For retarder, general powder type for Portland cement was used. Main components of retarder are calcium carbonate and methylcellulose.

After the formation of concrete layers, these bridging materials can be simply applied on top of the concrete layers using a different nose which can spread the materials.

The splitting tensile test was also implemented using Ø100 × 200 mm cylindrical specimen according to ASTM C496 (2011), Reinhardt et al. (1986) and Rocco et al. (2001) for estimation of bond strength (tensile strength) of the interface [11,12,13]. 

For estimation of tensile (bond) strength of interface of two concrete layers, splitting tensile test was performed. However, increment of bond strength due to the addition of bridging material was ignored. Therefore, for this study, conservatively, it was assumed that the bridging material only contributed to the fracture energy and not to tensile strength. Tensile strength obtained from splitting tensile test is the maximum point of normal stress–crack opening bilinear curves, which is normally called Mode 1 fracture behavior of the concrete. 

## 4. Theoretical Background of Fracture Energy Test and Setup

**Figure 5:**
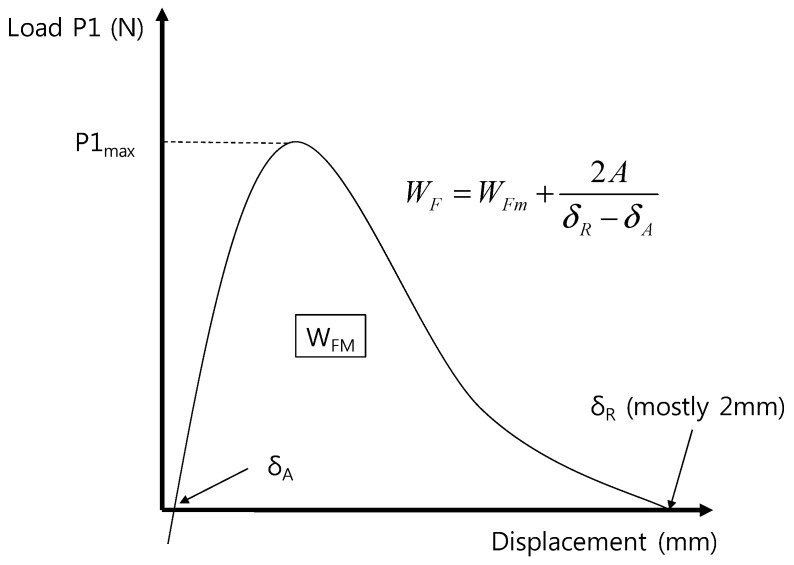
Measured and total works of fracture energy.

Experimental procedure to estimate the fracture energy of interface formed by using 3D printing technology was implemented by following the draft ASTM test standard (ACI 446 2009) for the fracture toughness presented by the ACI 446 committee in 2009 as well as the method by RILEM (2007) [10,11,14]. To conduct a quasi-static test that minimizes dynamic effects, low-speed test should be performed. The draft ASTM (2009) instructs to perform the experiments according to the constant rate of CMOD increase in the lower central notch of three-point bending test [11]. CMOD rate is not a set value and the CMOD rate of 3–5 min to reach the maximum load is recommended for the whole process of experiment. Appropriate CMOD rate for this study was calculated in line with the criteria and it is 0.0003 mm/s. The experiment needs to be continued at the rate until CMOD exceeds at least 2.0 mm. After 20 mm, fracture energy is not measured anymore; instead, fracture energy after 2.0 mm is estimated using slope of load–CMOD graph near point of 2.0 mm CMOD, as shown in Figure 5, where W_F_ is total work of fracture, Nmm (mJ); W_FM_ is measured work of fracture, Nmm (mJ); A is far tail constant, N-mm^2^; δ_R_ is load-point at the end of test, mm; and δ_A_ is CMOD at zero P_1_ for the raising part of curve, mm.

**Figure 6:**
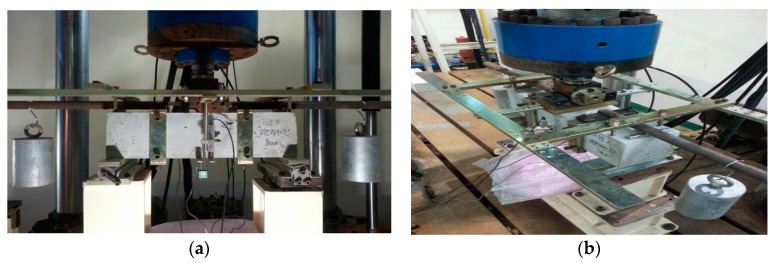
Three-point bending test set-up: (**a**) Front view; (**b**) Top perspective view

As shown in Figure 6, dead load from two cylindrical masses is working on the specimen under the principle of levers. Thus, the obtained load from load cell at 2 mm of CMOD also includes certain upward force from the dead load. To offset such an upward force generated by self-weight compensation, the whole graph must be offset upon test termination as in Figure 5 to make the load 0. In this process, δ_A_ is found. After the termination of 2 mm test, residual fracture energy remains in the specimen; however, the residual fracture energy after then is estimated through the equation in Figure 6. Given the characteristics of test conditions, load decreases asymptotically; thus, the test cannot be implemented until the load reaches 0. Therefore, at a certain point, the test should be terminated. The ACI 446 committee suggested 2 mm for the appropriate value [10]. The fracture energy values were different according to termination point (2 mm, 3 mm, 4 mm, and 5 mm) [9,10]. Further study will need to be performed in this regard and this present study applied the recommended value of 2 mm by the ACI 446 committee in the experimental procedure [10].

The far tail constant, A, in Figure 5, as explained above, is the value set to estimate the residual fracture energy after test termination.

**Figure 7:**
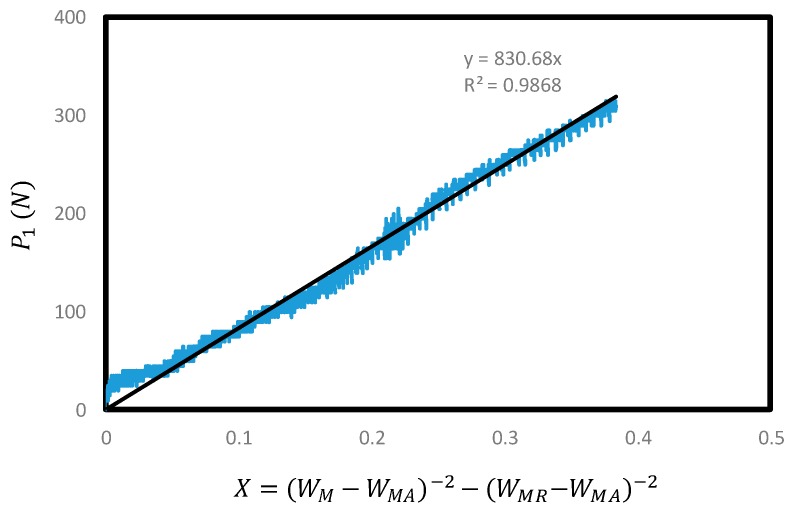
Estimation of far tail constant, *A*.

This is an example of an equation:

(1) $$P_{1}=X\left(A+K\right)$$

where P_1_, as mentioned above, represents the value of the existing load measurement value minus residual load from the cylindrical mass. The maximum P_1max_ load is shown in Figure 5 and P_1_ and *X* graphs are described in Figure 7. The quadratic equation with *X* is expressed in Equation (1) to calculate *A*. *X* is defined as presented by the *x*-axis in Figure 7. The area identified in the graph is based only on the data of <5% maximum load (P_1max_) in the whole curve. Thus, it was named as tail constant in this study. The ACI 446 committee named it (*A*) as far tail constant. *X* is the reciprocal of square of CMOD [10]. It can be related to load P_1_ with certain constant, *A*. The equation was formed based on the study findings of Petersson (1981) that the load (P)–displacement (*u*) graph decreased according to *u*^−2^ asymptotically [15]. Petersson (1981) presented that the load from kinematic equilibrium without consideration of rigid body’s dynamic effect was reverse proportional to displacement square as below [15]:

(2) $$P=\;\frac{bs}{4u^{2}}G_{F}\bar{w}.$$

where P represents load; *b* is specimen width; *s* is span; *u* is displacement (deflection); *G_F_* is fracture energy; and $\bar{w}$ is center of gravity on the area under the softening curve.

It is possible to estimate *A* values linearly using such a linear equation but the ACI draft (2007) instructs to use a quadratic equation to estimate *A* values. The Figure 7 below shows the graph utilized in *A* estimation. In the Figure 7 below, *W_M_* represents the measured CMOD value (mm); and *W_MR_* is the CMOD value (mm) measured upon test termination. *W_MA_* is the CMOD value (mm) when P_1_ is 0 in the incremental curve. Based on this, P_1_ and *X* values are shown using a diagram in Figure 7.

Figure 7 shows one of the cases found in the experiment. In this case, the tail constant (*A*) is deemed 830.7 (N-mm^2^). Thus, the estimated A value is substituted into the equation in Figure 7 to estimate energy value (*W_FM_*) and the estimated energy value is added to find the total energy (*W_F_*).

**Figure 8:**
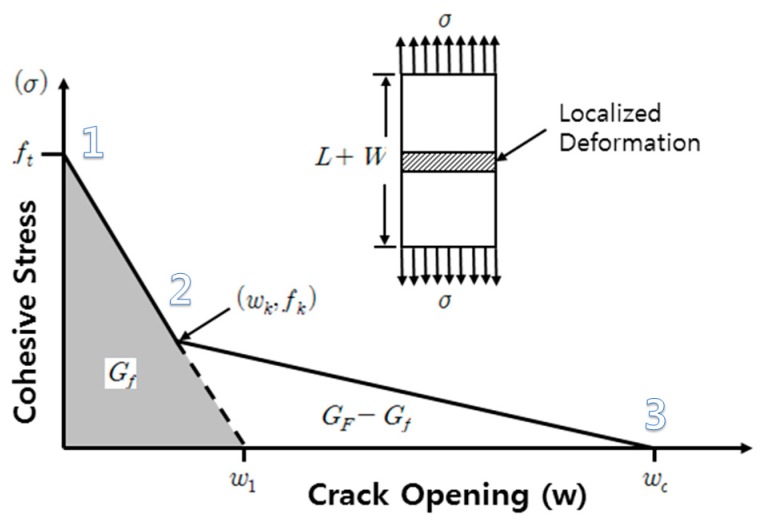
Bilinear approximation of the softening curve (Coronado and Lopez 2008).

In this study, about 3–5 h were required to measure the complete fracture energy of single specimen (min CMOD 2 mm). Specific details regarding test method, controversy surrounding the test method, conclusion and relevant theory can be found in Lee and Lopez (2014) and Gerstle (2010) [9,16]. The results measured in the three-point bending test and splitting tensile test can be finally determined as in Figure 8 based on the relationship between crack width and normal stress with bilinear approximation, which is useful for engineering applications. Here, *G_F_* is true fracture energy; and *G_f_*, is size effect fracture energy. The main properties of the bilinear approximation are the tensile strength (*f_t_*), the size-effect fracture-energy (*G_f_*), and the true fracture energy (*G_F_*). Detailed information can be found in Elices et al. (2002) [17]. In the graph below, point 1 (*f_t_*) can be estimated through splitting tensile test, and, afterwards, point 2 (crack width (*w_c_*) and stress at the kink point) and point 3 (point where stress become zero due to complete crack formation) can be estimated through fracture energy test. If a specimen in the size of this present study were utilized in an experiment, experimental results would be net of size effect [11,14,16,18,19,20,21,22,23].

The basic theory was presented by Planas et al. (1999) and Guinea et al. (1994), who developed the theory of Hillerborg (1979) [19,20,24].

Based on such experiment methods and theories, the present study analyzed changes in the final bilinear curve of concrete interfaces accumulated by 3D printing technology in various environmental conditions compared to normal plain concrete body.

## 5. Obtained Results from Fracture Energy Test (Fracture Toughness Test)

**Figure 9:**
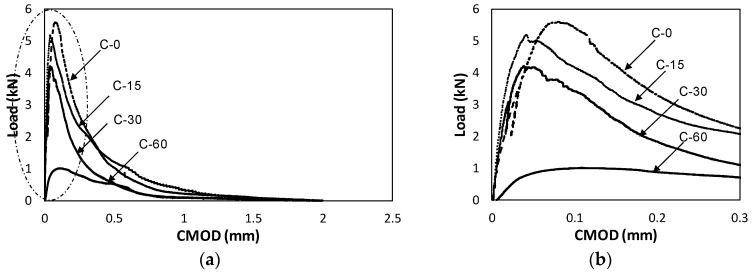
(**a**) Load–CMOD curves for C type specimens; and (**b**) enlarged graph.

The results of three-point facture energy test implemented using the above-explained procedure are represented in a form of load-CMOD graph, as shown in Figure 9. It should be noted that the obtained results could be sensitive to fresh concrete properties related to rheology. The data analysis method presented by ACI 446, Elices et al. (1992), Bazant and Planas (1997), RILEM (1990), Planas et al. (1999), and Lee and Lopez (2014) was employed to determine the final bilinear stress–crack opening curve depending upon the elapsed time to meet a new layer [10,20,22,25,26]. To determine such stress–crack opening curve, the load–CMOD graphs in Figure 9 should be analyzed first. All of the load–CMOD specimens were found to undergo maximum load decrease in the load–CMOD graph with increase in panel removal time (elapsed time to meet a new layer). Excluding the C-60 specimen, the three other specimens showed similar maximum loads, as shown in Figure 9. It was expected that the longer the time elapsed to contact the accumulated concrete layer with existing concrete layer, the larger reduction in maximum load was. Despite increase in the plate removing time, the maximum loads for 0 min, 15 min and 30 min were similar. However, for C-60 (60 min), a large decrease in maximum load was observed. Even though the maximum load could be controlled by notch area aggregate arrangement and any other heterogeneous state near the notch tip, a large reduction factor could be obtained from fracture energy test of C-60 specimens. From the conducted experiments, the initial and final setting time of the concrete was 90 and 220 min, respectively. Therefore, it can be assumed that the bonding of interface between two concrete layers before initial setting time might have been degraded.

**Figure 10:**
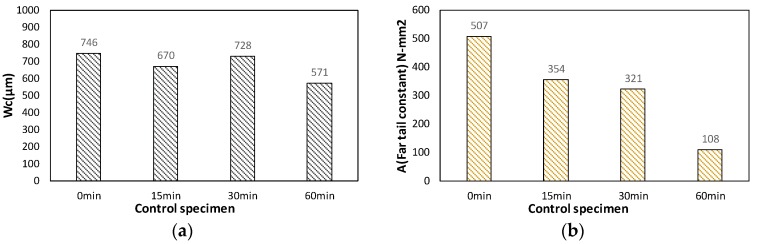
(**a**) Obtained results for far tail constant, *A*; and (**b**) critical crack opening displacement, *Wc*.

Figure 10a shows critical crack opening displacement (*w_c_*) from various conditions. The C-0 specimen was found to have 746 μm. For the S-60 specimen, *w_c_* was recorded as 571 μm, which demonstrated that the tendency of maximum crack width decreased as far as 77% of the maximum value with respect to the concrete with 0-min plate removing time (regular concrete). Figure 10b shows far tail constant (*A*) depending on the various elapsed times. The far tail constant, as explained above, is to estimate additional fracture energy that was not measured in this experiment after its termination at 2 mm of CMOD. Therefore, when the far tail constant becomes smaller, the final fracture energy drops. The far-tail constant, *A*, of plain concrete (C-0) was found to be 506.9 N-mm^2^, which is the maximum value. On the other hand, C-60 specimen recorded 108 N-mm^2^, showing a huge fall to 21.3% of the maximum value of plain concrete.

The experiment found the longer the elapsed time to meet a new layer (accumulation time), the smaller the maximum crack width (*w_c_*) and far tail constant (*A*). This might cause structural integrity reduction and structural performance decrease, particularly shear capacity.

**Figure 11:**
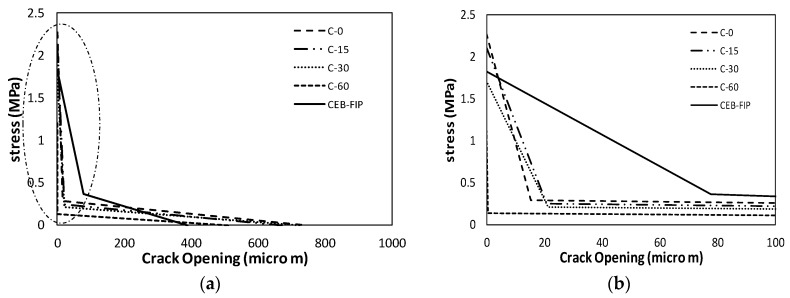
(**a**) Bilinear approximation of the softening curve for C type specimens; and (**b**) enlarged graph.

**Figure 12:**
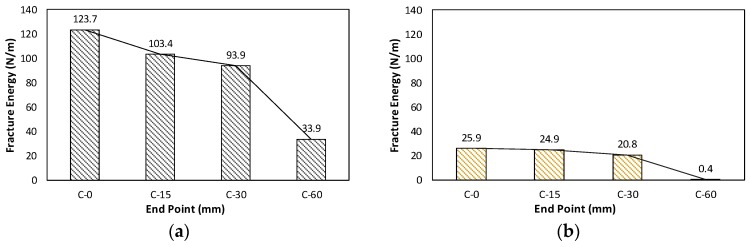
Comparison of *G_F_*, *G_f_*: (**a**) true fracture energy (*G_F_*); and (**b**) size effect fracture energy (*G_f_*).

The final bilinear stress-crack opening displacement is shown in Figure 11 with a bilinear graph based on CEB-FIP (2010) [23]. The calculated true fracture energy (*G_F_*) and size effect fracture energy (*G_f_*) have been compared in Figure 12 and Table 2. Both true fracture energy and size effect fracture energy showed decrease in energy with elapsed time to meet a new layer. For C-15 and C-30, 16.4% and 24.1% of fracture energy decreased when compared to the C-0 specimen. However, for C-60, 72.6% decrease in fracture energy was observed.

On the other hand, size effect fracture energy for C-15 and C-30 showed a small decreased fraction of the fracture energy. At 15 and 30 min, 3.86% and 19.7% decremented fraction of fracture energy was observed. However, for C-60, 98.46% decrease in energy was observed which was a large fraction compared to C-15 and C-30.

**Figure 13:**
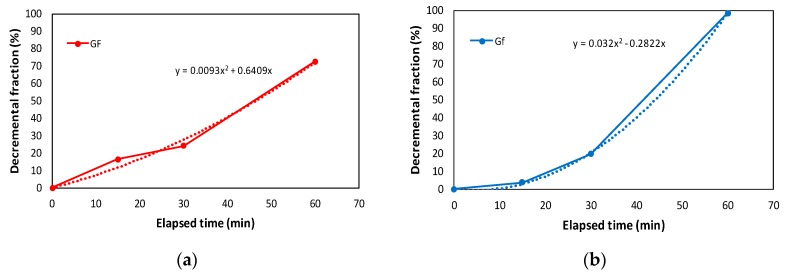
Elapsed time versus decremented fraction of fracture energy: (**a**) true fracture energy (*G_F_*); and (**b**) size effect fracture energy (*G_f_*).

Therefore, the relation between elapsed time and decremented fraction of true fracture energy was found to be linear, as shown in Figure 13a. However, the relation between size effect fracture energy and elapsed time was found to be nonlinear and was well fitted with quadratic equation, as shown in Figure 13b.

The result is deemed because the longer the accumulation time at the concrete layer interface, the harder the lower layer became to undermine its integrity with the upper layer. Initially, integrity reduction was expected to be remarkable if the interface formation time exceeded the initial concrete set time. However, in this study, huge reduction in fracture energy was found within 1 h when the initial concrete set time was longer than 1 h (90 min). These experimental observations show it is important to accumulate new layer far earlier than the initial concrete set time if the concrete interfaces would be accumulated by 3D printing technology.

**Figure 14:**
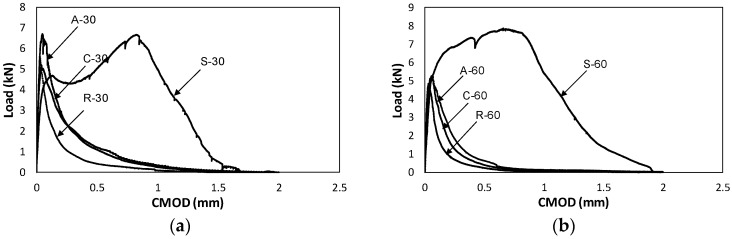
Comparison of Load–CMOD curves: (**a**) elapsed time to meet new layer (30 min); and (**b**) elapsed time to meet new layer (60 min).

Since fracture energy decreased with increased elapsed time, some bridge materials such as aggregates, retarders and steel fibers were considered, as previously mentioned. The obtained load–CMOD data are shown in Figure 14. Specific test plan regarding application of bridging materials has already been listed in Table 1.

For the S type specimen, increased fracture energy was significant (653.8%) and a procedure for obtaining stress–crack opening of steel fiber reinforced concrete should follow other experimental standards, such as RILEM TC 162-TDF [27]. The obtained fracture energies for S-30 and S-60 types were 328.0 N/m and 286.7 N/m, respectively. Therefore, by applying steel fibers at interfaces, better structural performances can be expected and it was concluded that degraded interface material character can be improved successfully.

**Figure 15:**
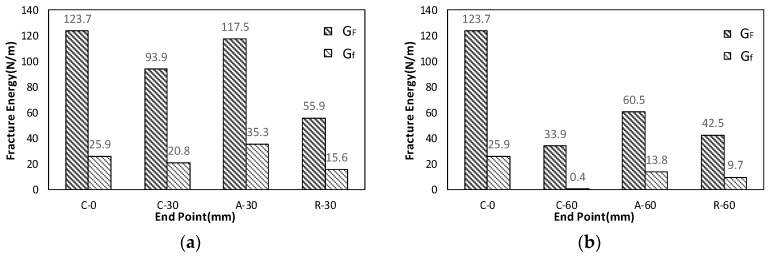
Comparison of *G_F_* and *G_f_*: (**a**) C-0, C-30, A-30 and R-30; and (**b**) C-0, C-60, A-60 and R-60.

Final true and size effect fracture energies for A and R types were compared with others, as shown in Table 3 and Figure 15. According to the summarized results shown in Table 3, when bridging materials were used, fracture energy such as A-30 increased up to the original level (95.0%) of fracture energy (C-0), indicating that the aggregate prevented decrease in facture energy successfully within elapsed time less or equal to 30 min. However, the retarders did not prevent the decrease in fracture energy successfully; instead, smaller fracture energy (55.9 N/m) was observed compared to the control type (C-30, 93.9 N/m).

To sum up, when steel fiber, aggregates and retarder were utilized, the fracture energy values were 328%, 95.0% and 45.2%, respectively when C-0 was set as 100%. This indicated that improved structural integrity with multiple interfaces could be achieved from bridging materials such as steel fiber and aggregates if the elapsed time to meet new layer is less than 30 min. However, if the elapsed time exceed 60 min, only steel fiber can maintain the original fracture energy under time effect. These final values of the obtained fracture energies of interfaces were used as input data of contact surface of Vector 2 development model presented in the next section.

## 6. Estimation of Reduction of Shear Strength Due to Addition of Interfaces

**Figure 16:**
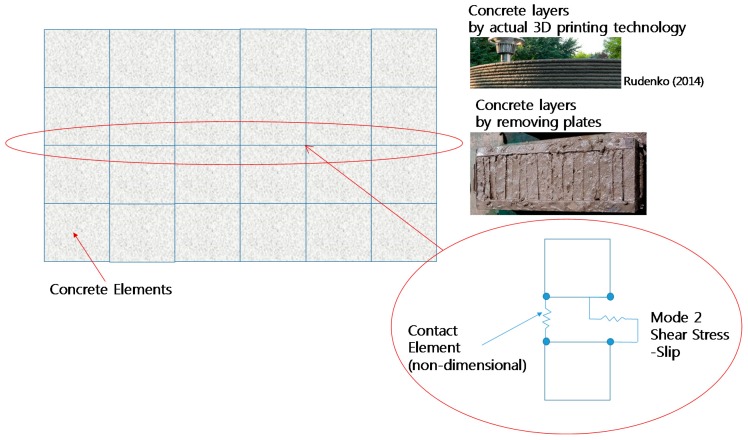
Modeling of interface between two concrete layers.

Based on measured fracture energy of the interface between concrete layers, reduction in shear strengths of various RC beams with multiple interfaces were estimated. RC beam with multiple interfaces could be also regarded as 3D printed RC beam, as shown in Figure 16. The basic material models selected for this study are listed in Table 4.

For the pre-peak response of concrete, Hognestad curves for the compression behavior of concrete was utilized and for the post-peak response of the concrete, modified Park and Kent models were used. For accurately estimating shear strength, modified compression field theory (MCFT) was considered. Since MCFT consider the cracked concrete subject to shear, it is appropriate for this study. In the present study, Vecchio’s 1992-A model was selected and this model was developed based on the results of extensive panel tests conducted by Vecchio and other researchers [28,29,30]. By using this model, overestimation of softening effects can be avoided when the principal tensile strains are significant. To model the tensile behavior of the concrete, the final forms of the stress–crack opening bilinear curves measured were used (see Figure 11).

For rebar, elasto-plastic behavior with hardening effects was considered. Truss elements were used for the rebars. In our study, smeared reinforcement was not considered. Instead, the bar elements for the rebar were considered separately.

For modeling the interface between the layers of concrete, contact elements were used. A contact element is a non-dimensional element and it needs two nodes at same location. For tangential behavior, Mode II fractured behavior such as shear stress–slip was used as the input parameter. Since Mode I fracture tests were conducted and found decremented fracture energy depending on elapsed time in this study, reduction of Mode II fracture energy was estimated based on reduction of Mode I fracture energy (normal stress–crack opening) obtained from fracture energy tests.

Detailed material behavior and MCFT implemented concrete models can be found in Wong et al. (2013) [31].

The selected element size for the concrete and reinforcement was 10 mm. According to Bazant (1986), an element of size two to three times larger than the maximum aggregate size is recommended for the modeling of cohesive cracking [32]. In the current study, the maximum aggregate size was 20 mm, therefore 50 mm would be adequate element size according to Bazant’s recommendation (1986) [32]. However, if 50 mm element size were used, the model could become too coarse to accurately predict the behavior of crack formation. Therefore, an element size of 10 mm using plane stress elements with aspect ratios of approximately 1.0 was used to refine the model. For effective computational time, a symmetric model was used. One node was selected as a support, where boundary condition for the support was restraint only in vertical direction, and horizontal displacement was allowed. The rebar was embedded into the concrete body as the nodes were shared with the concrete.

Monotonic loading was applied to the loading point with 1.0 kN increment. Displacement-based finite element method was used for Vector 2. Convergence was determined based on displacement values, with limit set to 1.00001. Weighted average displacement was used for the convergence criteria.

## 7. Shear Stress–Slip for Contact Elements

**Figure 17:**
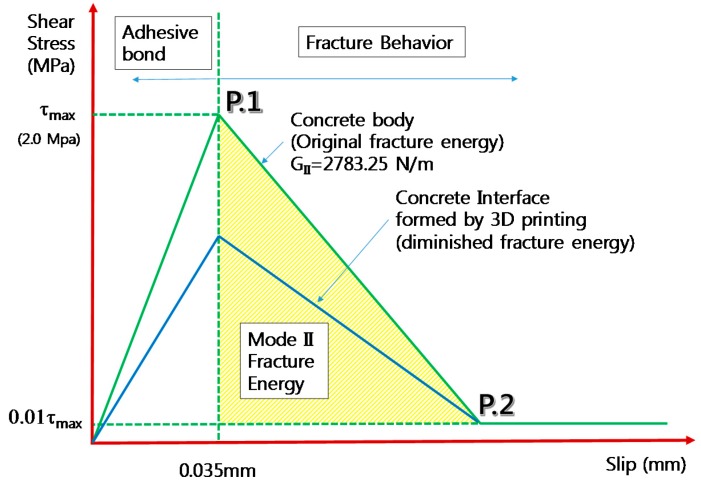
Shear stress–slip curve for contact elements in Vector 2.

For interfaces between two concrete layers formed before initial setting, contact elements were used, as previously described, and between two contact surfaces, shear stress–slip relationship (Figure 17) were entered as contact properties in Vector 2 program. First, when there was no reduction in contact properties, normal shear stress–slip curves, which were estimated from previous studies, were used. The maximum shear stress of P.1, shown in Figure 17, was estimated from the equation of CEB-FIP model code (2010), as shown below [23]. The first term was related to adhesive bond strength, while the second term was related to the frictional behavior, and the last term was related to the dowel action.

$$\tau_{u}=\tau_{c}+\mu \left(\rho \cdot k\cdot f_{y}+\sigma_{n}\right)+\alpha \cdot \rho \cdot \sqrt{f_{y}\cdot f_{cc}}$$

where $\tau_{u}$ represents ultimate shear strength of the concrete to concrete interface load; $\tau_{u\;}$ is frictional coefficient; $f_{y}$ is yield strength of the rebar; ρ is reinforcement ratio; k is interaction factor 1; α is interaction factor 2; and $f_{cc}$ is compressive strength of concrete under uniaxial stress.

Shear stress was estimated by adding frictional effect from dowel action as well as applied compressive stress to the adhesive bond stress of the interface. However, in the current study, frictional effect was ignored since analyses were conducted for the beam without stirrups and compressive stress acting at the interfaces. Therefore, adhesive bond stress was only considered and the adhesive stress is the maximum stress in Figure 17. The maximum shear stress (P.1 in Figure 17) for plain concrete was estimated as 2.0 MPa under rough interface condition of CEB-FIP (2010) [23]. The interface formed by 3D printing technology was considered better or comparable bonding condition to the sand blasted condition, since the interface is normally formed before the initial setting time of the concrete.

The slip at maximum shear stress in P.1 (see Figure 17) was estimated from Casal (2011) [33]. In the present study, the maximum value of adhesion was achieved at values of slip between 0.02 and 0.05 mm. In this study, 0.035 mm was selected as slip at maximum shear stress. The Mode II fracture energy after maximum stress was estimated following the report of Shilang and Reinhardt (2005) [34]. The Mode II fracture energy is the shaded area under the curve shown in Figure 17. Therefore, P.2 could be determined after estimation of Mode II fracture energy. For sound concrete interfaces of concrete body, Mode II fracture energy was assumed to be 2783 N/m referring to Shilang and Reinhardt’s (2005) recommendation [34]. Decreased ratio of Mode II fracture energy due to environmental conditions such as elapsed time was predicted based on obtained results from Mode I fracture tests. For example, if Mode I fracture energy decreased to 70% of original energy due to environmental effect such as the elapsed time compared to original concrete body, it was assumed that Mode II fracture energy would also decrease to 70% of the original energy.

For the stress at P.2, it was assumed that 1% of the residual shear stress was present even after complete fracture of interface. By assuming that there was residual stress after complete fracture, stable computation and convergence rate was obtained.

## 8. Model Verification

**Figure 18:**
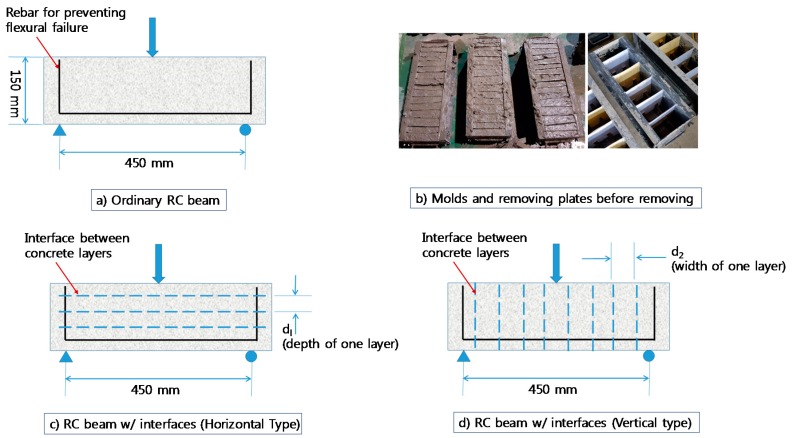
Two types of specimen for model verification: (**a**) Ordinary RC beam ; (**b**) Molds and removing plates before removing; (**c**) RC beam w/interfaces (Horizontal Type); (**d**) RC beam w/interfaces (Vertical Type).

To verify the selected model for estimating shear strength of the RC beam, specimens shown in Figure 18 were made. Ordinary RC beam and layered RC beam with multiple interfaces were made using several removing plates. Two D10 rebars were embedded. The interface was first made as shown in Figure 18b, and after 60 min the plates were removed to generate interfaces between the new layers and old layer before setting the initial time. Depth (d_1_) and width (d_2_) of layers were 30 mm and 35 mm, respectively.

**Figure 19:**
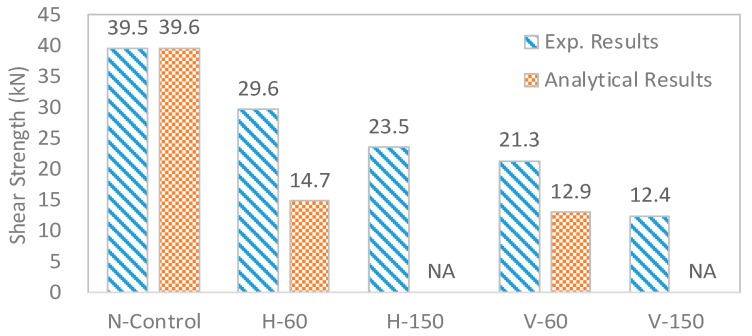
Obtained results of shear strength.

The obtained results are shown in Figure 19. Normal control RC beam (N-Control) showed maximum load of 78.9 kN (shear strength: 39.5 kN). The layered beams showed lower failure loads compared to the N-Control as expected. The horizontal types (H-60 and H-150) with elapsed time 60 min and 150 min showed 59.2 kN and 46.9 kN, respectively, indicating that strength decrements were 24.9% and 40.6% from the horizontal interfaces. Likewise, the vertical types (V-60 and V-150) with elapsed time 60 min and 150 min showed 42.6 kN and 24.7 kN, respectively, indicating that the strength decrements were 46.0% and 68.7% from the vertical interfaces. Therefore, strength decrement was higher for the vertical type than the horizontal type, while the area of interface for vertical type was approximately equivalent to the horizontal type. The horizontal types showed higher shear strength than the vertical types since the superimposed load (self-weight of each layer) of the horizontal type was more effectively applied at the interfaces during curing of the concrete than that of the vertical type. Model verification was done using the experimental data.

As previously explained, interface property of the layers was designed using Mode II fracture behavior employing Vector 2 program. As shown in Figure 19, considering reduction in fracture energy, shear strength decrement was predicted. Since the measured fracture energy was Mode I, it was assumed that ratio of reduction in fracture energy in Mode II is same as reduction in fracture energy in Mode I. From the fracture energy under the curve of bond–slip behavior of sound interface condition (100%), decreased fracture energy ratio of interfaces was estimated using the experimental fracture energy. For example, the obtained experimental fracture energy of interface was 72.6% of the original fracture energy of normal concrete, as shown in Figure 8. Therefore, 72.6% fracture energy was considered at the interfaces of layered beam. The obtained results are shown in Figure 8. In the case of normal concrete, good agreement between experimental and analytical results was obtained. However, in the case of layered concrete beams, such as H-60 and V-60, shear strength decrements due to the degraded interfaces could not be estimated well. H-150 and V-150 could not be predicted since the fracture energy with elapsed time 150 min were not measured. It was thought that this difference came from different behaviors between Mode I and Mode II fracture energies. Even though Mode I fracture energy was diminished up to 72.6%, Mode II fracture energy of the concrete did not change as Mode I.

**Figure 20:**
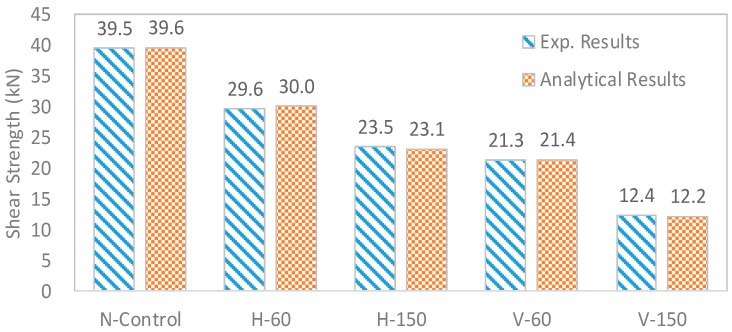
Obtained results of shear strength.

Since the developed model used Mode II fracture for failure of the interfaces, decrement ratio of Mode II fracture energy was predicted by performing several reverse analyses, by adjusting the level of Mode II fracture energy at interfaces, the calculated shear strengths were compared with experimental data and the cases which showed the smallest error were selected. The selected levels of Mode II fracture energy for H-60, H-150, V-60 and H-150 were 75%, 45%, 50% and 26% of original Mode II fracture energy, respectively, and the obtained results are shown in Figure 20.

**Figure 21:**
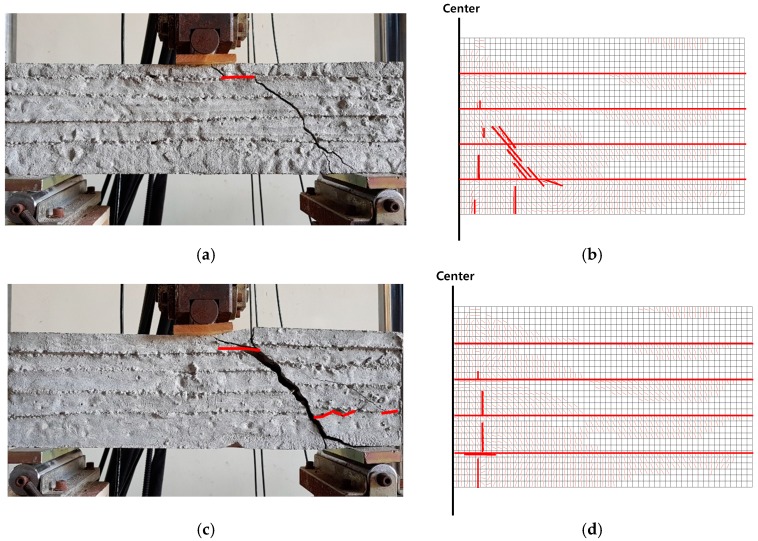

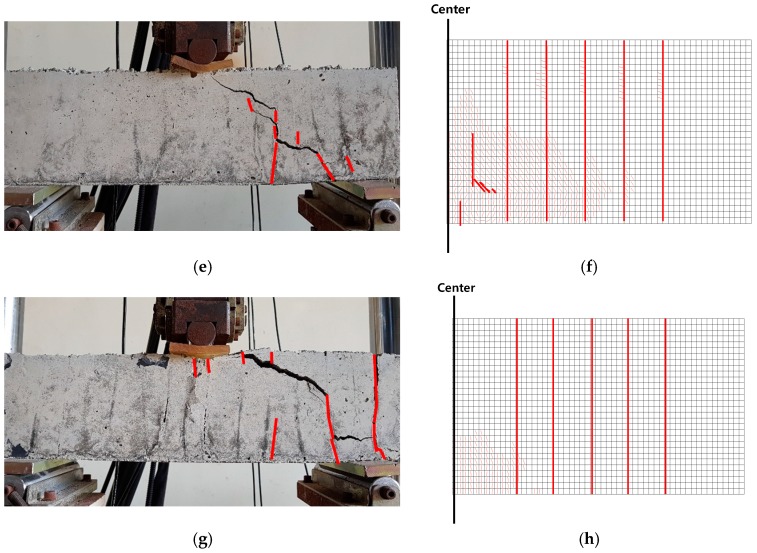
Crack patterns from experiments and analysis: (**a**) H-60 (experiment); (**b**) H-60 (analysis, scale 20); (**c**) H-150 (experiment); (**d**) H-150 (analysis, scale 20); (**e**) V-60 (experiment); (**f**) V-60 (analysis, scale 20); (**g**) V-150 (experiment); and (**h**) V-150 (analysis, scale 20).

Since the a/d was about 1.8 and rebar was used to reinforce the flexural capacity, the shear failure modes were obtained from all beams as intended. Crack patterns from experiments and analysis were compared, as shown in Figure 21. In the figure, it can be found that the interface with diminished fracture energy affected the crack pattern, and for layered RC beam, the cracks were also formed at the interfaces. In Figure 21, cracks formed at interface were indicated by red line. However, overall crack pattern observed from layered concrete beam are still similar with that of ordinary RC beam under shear. This indicates that even though the weakest link is located in interface, major cracks could be formed in interlayer body between interfaces.

## 9. Obtained Results Regarding Shear Strength Reduction

To estimate the reduction in shear strength of the general RC beam with interfaces, previous shear tests on RC beams from other research work were collected, and specimens designed by Xie et al. (1994), Yang et al. (2007) and Mihaylov et al. (2010) were modeled [35,36,37]. Particularly, in the present study, short beams, viz. a/d between 1.0 and 2.0, without stirrups were considered in order to see the pure shear strength of the concrete with dowel action from tension rebar. Therefore, no additional effects from stirrups on shear strength was considered in this study, indicating that the obtained shear strength consisted of concrete shear strength with small aggregate interlocking effect and dowel action with no stirrup condition. Finally, shear strengths of the RC beam with and without interfaces were predicted and compared with experimental results.

For ordinary RC beam without multiple interfaces, the average percent difference between experiments and analysis was 8.67% and the developed model predicted the shear capacities of the RC beam very well. After verification, it was assumed that the selected beam was made by 3D printing technology, resulting in the formation of multiple interfaces. The previously found level of Mode II fracture energy (75%, 45%, 50% and 26%) were next applied to the beams with horizontal and vertical interfaces with 60 min and 150 min elapsed time. The obtained results for prediction of shear strength as well as other important parameters are summarized in Table 4. For horizontal interfaces (H type) with 60 and 150 min elapsed time, the average shear strength reductions were predicted to be 33% and 48%, respectively. Likewise, for vertical interfaces (V type) with 60 and 150 min elapsed time, the average shear strength reductions were predicted as 65% and 80%, respectively.

**Figure 22:**
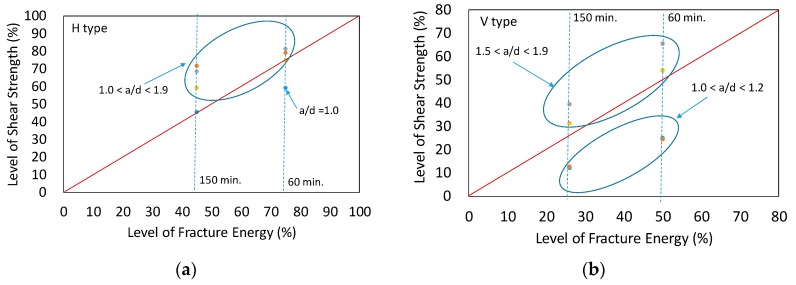
Fracture energy vs Shear strength: (**a**) horizontal type; and (**b**) vertical type.

Based on the obtained results shown in Table 5, the decreased fracture energy affected the shear strength of RC beam with multiple interfaces, as seen in Table 5. To find a relationship between fracture energy and shear strength of RC beam with multiple interfaces, we defined the level of fracture energy (%) as decreased fracture energy divided by original fracture energy. Likewise, the level of shear strength (%) was defined as decreased shear strength due to the existence of interfaces with time effects divided by shear strength from normal RC beam. The level of fracture energy (%) versus the level of shear strength (%) is shown in Figure 22. For horizontal types, the shear strength reductions were smaller than the reduction in fracture energy except one case (a specimen of Xie et al. (1994) with an assumption of 60 min elapsed time) [35]. Therefore, for horizontal types with less than 60 min elapsed time, it can be conservatively concluded that the level of shear strength reduction is equivalent to the reduction in fracture energy. Reverse analysis showed that, in 60 min, 75% level of fracture energy remained at the interfaces. Hence, 75% shear capacity of layered RC beam with horizontal interfaces compared to the normal RC beam was expected. However, for vertical types, the shear strength reductions were smaller or larger than the reduction in fracture energy depending on a/d, as shown in Figure 22b. For shorter beams (1.0 < a/d < 1.2), reduction in shear strength was more significant compared to the other beams (1.5 < a/d < 1.9). Therefore, short beams with a/d range less than 1.2 will undergo large reduction if it is made by 3D technology. However, other beams with a/d range more than 1.5 showed less reduction in shear strength compared to the reduction in fracture energy.

## 10. Proposed Shear Reduction Factor for RC Beam with Interfaces Formed by 3D Printing Technology

Based on the obtained results shown in Figure 22, shear reduction factors for RC beam with multiple interfaces formed by 3D printing technology were proposed, as shown in Table 6. Depending on the interface type, such as horizontal and vertical types, and elapsed time, such as 60 and 150 min, the reduction factors are calculated. Elapsed time is normalized by initial setting time. It should be noted that the following factors could be used for estimating shear strength of short RC beam (1.0 < a/d < 2.0) with dowel action due to the existence of rebars, but without any stirrups. Interfaces were formed before final setting time (220 min) of the concrete. The proposed shear reduction factors were dependent on many environmental conditions, such as humidity, temperatures and cement properties. Further experimental studies are necessary for accurately selecting the reduction factor.

$$V_{c\_layered}=\alpha V_{c}$$

where $V_{c}$ is the Shear strength of RC beam without stirrup; α is the Shear reduction factor (refer to Table 6); and $V_{c\_layered}$ is the Shear strength of RC beam with interfaces.

## 11. Conclusions

From the results of concrete fracture energy tests and developed shear strength model, the following conclusions can be derived.

1. The elapsed time for the new layers to form concrete structures reduces the total fracture energy significantly (72.6%) especially when the elapsed time (60 min) is shorter than the initial setting time (90 min) of the concrete.
2. Bridging materials such as steel fibers and aggregates improved the overall structural integrity by increasing the fracture energy at interface between two layers. The maximum improvement was realized on addition of steel fibers, as expected. Aggregates prevented any reduction in fracture energy due to the elapsed time and finally could maintain the original level of fracture energy. However, retarders did not successfully prevent decrease in fracture energy with increased elapsed time to form a new layer.
3. A model to predict the shear strength of RC beam was developed using Vector 2 software following the MCFT theory. Model verification was conducted and it was found that the shear strength of different scales of the RC beam could be satisfactorily predicted with approximately less than 10% margin.
4. By using the newly developed model, the reduction in shear strength of RC beams with multiple interfaces and without stirrups were estimated from the results of the fracture test. It was found that, at the initial setting time of the concrete, the fracture energy diminished, which affected the overall behavior of the general RC beam. Finally, the average reduction in shear strength for horizontal interfaces at 67% of initial setting time was 33%. Likewise, the reduction in shear strength for vertical interfaces at 67% of initial setting time was 65%.
5. Shear reduction factors depending on span to depth ratio (a/d) and elapsed time to meet new layers were also proposed. The vertical interfaces showed larger reduction in shear than the horizontal ones.
6. In the present study, the reduction of Mode II fracture energy at interface of layered concrete structure was estimated by reserve analysis using the newly developed model. In future studies, Mode II fracture energy of interfaces will be measured.

## Figures and Tables

**Table 1:** Test plan for fracture energy of interfaces.

Specimen	Bridging Materials	Removing Plate Time (Min)
C-0	NA (Control Specimens)	0
C-15		15
C-30		30
C-60		60
A-30	Aggregate (8 mg/mm^2^) *	30
A-60		60
R-30	Retarder agent (1.75 g/1 kg Concrete) *	30
R-60		60
S-30	Steel fiber	30
S-60	(1%) *	60

* Numbers in parenthesis show amount of bridging material used per unit area, unit weight and unit volume.

**Table 2:** Results of fracture tests for (C type of specimens).

Specimen	Bridging Materials	Plate Removing Time (Min)	*Wc* (µm)	*A* (Far Tail Constant, N-mm^2^)	Size Effect Fracture Energy (N/m)	True Fracture Energy (N/m)
C-0	NA (Control Specimens)	0	746	507	25.9	123.7
C-15		15	670	354	24.9	103.4
C-30		30	728	321	20.8	93.9
C-60		60	571	108	0.4	33.9

**Table 3:** All Results obtained from fracture energy test.

Specimen	Bridging Materials	Plate Removing Time (Min)	Size Effect Fracture Energy (N/m)	True Fracture Energy (N/m)
C-0	NA (Control Specimens)	0	25.9	123.7
C-15		15	24.9	103.4
C-30		30	20.8	93.9
C-60		60	0.4	33.9
A-30	Aggregate (8 mg/mm^2^)	30	35.3	117.5
A-60		60	13.8	60.5
R-30	Retarder agent (1.75 g/1 kg Concrete)	30	15.6	55.9
R-60		60	9.7	42.5
S-30	Steel fiber (1%)	30	-	328.0
S-60		60	-	286.7

**Table 4:** Selected material models for estimating shear strength.

Material	Selected Models	Notes
Concrete	Hognestad	Prepeak
	Modified Park and Hent	Post-peak
	Vecchio 1992-A	Softening Compression
	Strain based custom input	Softening Tension
	Kupfer/Richardt	Confined strength
	Variable Kupfer	Dilation
	Mohr-Coulomb (stress)	Cracking Criterion
	DSFM/MCFT	Crack stress calculation
	Agg./2.5 maximum crack width	Crack width check
	Walraven	Crack slip
Steel Rebar	Bauschinger effect	Hysteretic
	Tassios (crack slip)	Dowel action
	Refined Dhakal-Maekawa	Buckling
Interface between layers	Bilinear Shear Stress–Slip Behavior	Contact material properties

**Table 5:** Obtained results for predicting shear strength of normal and layered RC beams.

Authors	Test Specimen	a/d	*f_c_*′ (MPa)	*G_F_* (N/m)	*f_t_* (MPa)	Rebar in Tension Zone	Comp. Rebar	Normalized Elapsed Time (%)	Exp. V_u_ (kN)	Predicted V_u_ (kN)	Differences (%)
Yuliang et al. (1994)	Ordinary RC beam	1	46.9	150	3.9	2@D19	NA	NA	156	140	10.3
	RC beam w/interfaces (Horizontal)	1	46.9	150	3.9	2@D19	NA	67	NA	83	NA
	RC beam w/interfaces (Horizontal)	1	46.9	150	3.9	2@D19	NA	167	NA	64	NA
	RC beam w/interfaces (Vertical)	1	46.9	150	3.9	2@D19	NA	67	NA	35	NA
	RC beam w/interfaces (Vertical)	1	46.9	150	3.9	2@D19	NA	167	NA	17	NA
Yang et al. (2007)	Ordinary RC beam	1.17	32.1	142	3	3@D19	3@D19	NA	440	410.5	6.7
	RC beam w/interfaces (Horizontal)	1.17	32.1	142	3	3@D19	3@D19	67	NA	326	NA
	RC beam w/interfaces (Horizontal)	1.17	32.1	142	3	3@D19	3@D19	167	NA	229	NA
	RC beam w/interfaces (Vertical)	1.17	32.1	142	3	3@D19	3@D19	67	NA	101	NA
	RC beam w/interfaces (Vertical)	1.17	32.1	142	3	3@D19	3@D19	167	NA	52	NA
Boyan et al. (2010)	Ordinary RC beam	1.55	34.2	143	3.1	6@D25	6@D25	NA	710	646	9
	RC beam w/interfaces (Horizontal)	1.55	34.2	143	3.1	6@D25	6@D25	67	NA	527	NA
	RC beam w/interfaces (Horizontal)	1.55	34.2	143	3.1	6@D25	6@D25	167	NA	443	NA
	RC beam w/interfaces (Vertical)	1.55	34.2	143	3.1	6@D25	6@D25	67	NA	423	NA
	RC beam w/interfaces (Vertical)	1.55	34.2	143	3.1	6@D25	6@D25	167	NA	255	NA

**Table 6:** Proposed shear reduction factors.

Interface Type	Normalized Elapsed Time (%)	Range of a/d	Shear Reduction Factor (a)
Horizontal	67	1.2 < a/d < 1.9	0.75
	167	1.0 < a/d < 1.9	0.45
Vertical	67	1.5 < a/d < 1.9	0.5
	167	1.5 < a/d < 1.9	0.26
	67	1.0 < a/d < 1.2	0.25
	167	1.0 < a/d < 1.2	0.12

Normalized elapsed time: Elapsed time divided by initial setting time (90 min) of the concrete.
